# Cyclo‐Octasulfur Crystals as Light‐Controlled Molecular Muscles

**DOI:** 10.1002/anie.202506269

**Published:** 2025-07-01

**Authors:** Enrique Solano‐Rodríguez, Qi Sun, Jean‐Luc Brédas, Veaceslav Coropceanu, Beatriz Jurado‐Sánchez, Alberto Escarpa

**Affiliations:** ^1^ Department of Analytical Chemistry Physical Chemistry and Chemical Engineering Universidad de Alcala Alcala de Henares Madrid E‐28802 Spain; ^2^ Department of Chemistry and Biochemistry The University of Arizona Tucson Arizona 85721‐0041 USA; ^3^ Chemical Research Institute “Andres M. Del Río” Alcala de Henares Madrid E‐28802 Spain

**Keywords:** Inorganic actuators, Photo‐responsive, Sulfur crystals

## Abstract

Here, we describe the synthesis and photo‐responsive properties of 2D cyclo‐octasulfur microcrystals (α‐*S8* MCs), produced using a quick, simple, cost‐effective, and environmentally friendly hydrothermal method. Controlled 385‐nm irradiation of these crystals induces an immediate, reversible, and sustained bending effect. The time required for the crystals to return to their initial shape is significantly reduced when exposed to 475‐nm light, completing the entire excitation‐relaxation cycle in less than 2 s. Moreover, this process can be repeated up to 50 times as long as the crystals remain in water. The dependence of recovery time on light wavelength is rationalized qualitatively via highly correlated quantum‐chemical calculations of the absorption spectra of *S8* chains. The underlying mechanism involves a combination of ring‐to‐chain and chain‐to‐ring transformations: the breaking of α‐*S8* rings by 385‐nm radiation induces bending in the α‐*S8* MCs, while the excitation of the chains with 475‐nm light facilitates an accelerated recovery process, allowing the *S8* molecules to swiftly regain their ring shape. Thus, our study demonstrates that α‐*S8* MCs represent intelligent actuators as light‐controlled molecular muscles with the simplest inorganic composition reported to date.

## Graphical abstract

Bending and stretching effect provoked by the repeated application of 385‐nm radiation (purple) and 475‐nm radiation (blue) on sulfur microcrystals.

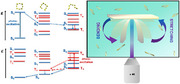

## Introduction

So‐called intelligent materials are those that undergo specific changes in their physicochemical properties in response to external stimuli. This trigger can be a mechanical stress (piezoelectric materials) or a change in temperature, pH, or light (photo‐responsive materials). Intelligent materials play an essential role, for instance, in the miniaturization of electronic devices, in efficiency improvements, or in remote‐controlled actions across a broad range of fields, including electronics, medicine, or energy.^[^ ^1^, ^2^, ^3^ ^]^ Among intelligent materials, photo‐responsive compounds are of particular interest. With only a light source, a chemical–physical change can be induced, which opens the door for easy remote‐controlled systems. Photo‐responsive materials encompass a diverse range, from silver salts used in the early development of photography to phase‐transition polymers designed for thermal energy harvesting.^[^ ^4^ ^]^ One of the main attributes sought in such systems is reversibility. Indeed, to develop a remote‐controlled system, it is desirable to induce a temporary change that can return to its initial state through a relaxation process.^[^ ^5^ ^]^ Even better, if the recovery can be triggered by a secondary stimulus, this can create a time‐independent and controllable on‐off system for a promising range of applications.^[^ ^6^, ^7^, ^8^, ^9^ ^]^

Photo‐responsive shape changes have been observed in organic materials, driven primarily by two mechanisms: photoisomerism and photothermal effects. In the case of photoisomerism, light absorption excites the molecules, inducing a structural transformation that generates an isomer of the original molecule. One example of this effect can be found in the ferroelectric compound *N*‐salicylidene‐2,3,4,5,6‐pentafluoroaniline, which undergoes a light‐triggered structural change through reversible photoisomerization between the *cis‐enol* and *trans‐keto* forms.^[^ ^10^ ^]^ On the other hand, photothermal mechanisms convert light energy into thermal energy. This effect provokes a structural and/or chemical change in the molecules. Photothermal processes can be seen, for example, in ferroelectric crystals derived from drying guanidinium nitrate, which undergo structural changes in their crystalline structure, resulting in shape change and deformation when they are subjected to heating and cooling processes.^[^ ^11^ ^]^ Such a photothermal mechanism has also been reported for (*S*)‐*N*‐3,5‐di‐*tert*‐butylsalicylidene‐1‐(1‐naphthyl)ethylamine in the *enol* form that undergoes an *enol‐keto* photoisomerization process.^[^ ^12^ ^]^ Incorporating photo‐melting azobenzene monomers into polymers creates a photo‐responsive polymer with UV–vis light‐controlled deformations due to changes in its molecular structure.^[^ ^13^ ^]^

However, while most photo‐responsive materials are organic, inorganic ones are much less common. One of the few reported examples is the crystals of [Co(NH_3_)_5_(NO_2_)]Cl(NO_3_) that can hop and move across surfaces under UV light due to internal polymerization, which generates mechanical stress within the crystal.^[^ ^14^ ^]^ Among inorganic compounds, sulfur stands out as a stable and abundant element that offers a compelling platform for functional material design. Following carbon, sulfur possesses the greatest number of known allotropes. The most common form is the α‐phase, an orthorhombic polymorph composed of repeating eight‐membered rings (*S8*). Upon heating to 368 K, α‐sulfur undergoes a phase transition to the β‐phase, which adopts a monoclinic structure. In its native α‐form, it is an insulating material with a low melting point (388 K), which remains very stable in water across a wide pH range.^[^ ^15^ ^]^ Sulfur also possesses photoactive properties; however, in most materials reported to date, it is part of a composite with other elements, which can mask its intrinsic properties and potential. Thus, while intelligent materials incorporating sulfur have been reported, pure sulfur has yet to be proven as a viable candidate for related applications.^[^ ^16^, ^17^, ^18^ ^]^ A few reports have indeed illustrated the on‐demand, light‐triggered control of sulfur‐based materials. For example, an α‐*S8* powder can be activated by irradiation with both UV and visible light and used for water splitting or the decomposition of organic molecules via the generation of OH radicals.^[^ ^19^ ^]^ In this context, the properties of sulfur have been exploited in photocatalysis as well as in environmental‐related and energy‐related applications.^[^ ^20^ ^]^ Elemental sulfur has also been utilized in an innovative polymerization process called inverse vulcanization,^[^ ^21^, ^22^ ^]^ enabling the resulting polymers to find applications in infrared optics, thermal imaging,^[^ ^23^, ^24^, ^25^ ^]^ energy storage,^[^ ^22^ ^]^ rubber vulcanization agents,^[^ ^26^ ^]^ self‐healing materials,^[^ ^27^, ^28^ ^]^ laser lithography, and erasable information storage.^[^ ^29^ ^]^

In this study, we obtained α‐*S8* microcrystals (α‐*S8* MCs) as a sub‐product of the hydrothermal synthesis of MoS_2_, using (NH_4_)_2_MoS_4_ as precursor. After isolation of the crystals, their characterization revealed that they consist of the pure, photo‐responsive α‐*S8* sulfur polymorph; the crystals bend under 385‐nm radiation and recover their original shape within 1 s when 475‐nm blue light is applied. Based on electronic‐structure calculations, we indicate that the bending effect arises from the structural transformations of *S8* molecules, specifically the conversion from *S8* rings to chains. Conversely, the recovery process corresponds to the restoration of the original ring structure through a chain‐to‐ring transformation. This reversible interconversion between ring and chain forms underlies the observed photo‐responsive properties of the system. To the best of our knowledge, the observation of such behavior in inorganic sulfur crystals is unprecedented.

## Results and Discussion

**Figure 1:**
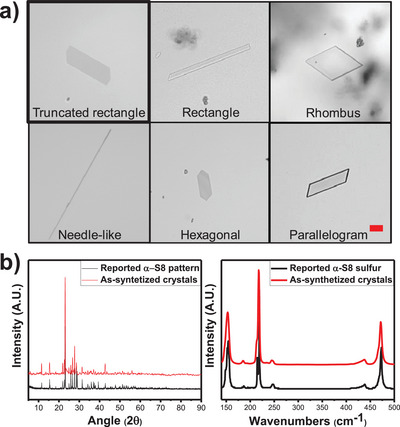
a) Optical microscopy images showing the different morphologies of the α‐*S8* MCs. Scale bars 10 µm. b) XRD pattern (left) of as‐synthetized α‐*S8* MCs (red) and reported α‐*S8* MCs (black), and Raman spectra (right) of as‐synthetized α‐*S8* MCs (red) and reported α‐*S8* sulfur (black).^[^ ^31^ ^]^

α‐*S8* MCs were obtained as a subproduct of the synthesis of MoS_2_ (see Scheme S1). Briefly, 20 mg of (NH_4_)_2_MoS_4_ were dissolved in 30 mL of deionized water, resulting in an orange solution. Then, 70 µL of hydrazine solution (35 wt % in water) were added and the resulting mix was put into a 50 mL stainless‐Teflon reactor. Hydrothermal treatment was carried out for 10 h at 110 °C. After this period, the original orange solution turned into a black dispersion of MoS_2_ flakes. The product was isolated via centrifugation (5000 RCF for 10 min). Black supernatant was collected and stored for 3 to 4 days. On the very first day, it is possible to observe a white powder on the bottom of the vial. According to previous studies^[^ ^30^ ^]^ and after elemental analysis (Figure S1), that powder corresponds to sulfur nanoparticles. During the next 2 to 3 days, a bottom‐up process takes place, assembling the sulfur particles themselves to give different crystalline structures. Once the α‐*S8* MCs have been formed, the initial black solution becomes colorless. α‐*S8* MCs appeared like shiny small flakes on the base of the container with different shapes, from rectangular to needle‐like shapes in the same synthesis (Figure 1a). The synthesis proved to be highly reproducible, with the truncated rectangle shape (Figure 1a, up left) always being the most prevalent (ranging from 90% to 95%) in the different batches of crystals obtained, with highly reproducible length (80 ± 30 µm) and width (28 ± 17) dimensions (*n* = 30).

After isolation, α‐*S8* MCs were studied using different characterization techniques. X‐ray powder diffraction (XRD) data display a crystalline pattern consistent with that reported for the α‐*S8* sulfur (Figure 1b, left) from RRUFF Database (RRUFF ID: R050006.1). The same conclusion can be drawn from the Raman spectroscopy results, as evidenced by the comparison between our data and the literature data (Figure 1b, right).^[^ ^31^ ^]^

**Figure 2:**
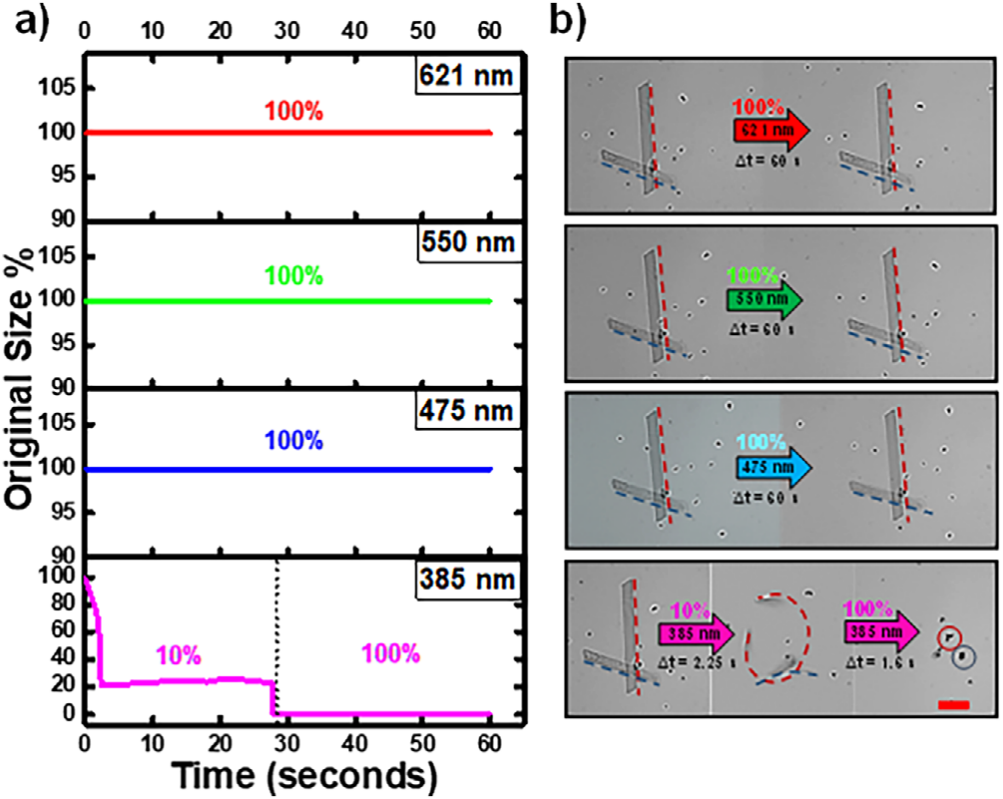
Size variation of α‐*S8* MCs under different wavelength radiation, a) expressed as percentages of the original size (see Figure S2), and b) α‐*S8* MCs images taking from Video S2 during the irradiation (red and blue dashed lines help to see the crystal size). All cases with the maximum power of the LEDs (100%) but in 385 nm radiation, first 10% and second 100% of power. Scale bar, 20 µm.

To study the photosensitive response, a drop of α‐*S8* MCs, dispersed in the same water solution as that of the synthesis, was placed on a glass slide. The α‐*S8* MCs were then irradiated using a 40× microscope lens to focus the light (Video S1). The α‐*S8* MCs were exposed to various wavelengths lights: 621, 550, 475, and 385 nm. The resulting data are presented in Figure 2 and Video S2.

When the α‐*S8* MCs are exposed to 621, 550, and 475 nm radiation, they do not show any reaction or modifications in shape or size, even when the radiation lasts for 1 min with 100% of the power of the LED. However, interestingly, if the same α‐*S8* MCs were exposed to 385‐nm radiation, even with 10% of the power (4.7 mW mm^−2^) is enough to immediately provoke a deformation by bending. After up to 28 s under this radiation, the power of the LED was increased to 100% (44 mW mm^−2^), resulting in a dramatic crush of the α‐*S8* MCs in only 2 s (see Video S2). No further changes were observed after the 30 s that the radiation was acting on the collapsed sulfur crystals after the crush.

Following these observations, the 385‐nm radiation was used for a more detailed study of the photosensitivity via modulation of its intensity. When the α‐*S8* MCs were exposed to 385‐nm radiation with an intensity of 10% of the original exposure, they bent along their transverse axis instead of being crushed. Switching off the radiation, and only with the presence of daylight, the original shape was recovered after 22 s. There were no other visible changes in the α‐*S8* MCs. This cycling experiment was carried out three times, taking in all three approximately the same time to recover to the original shape.

**Figure 3:**
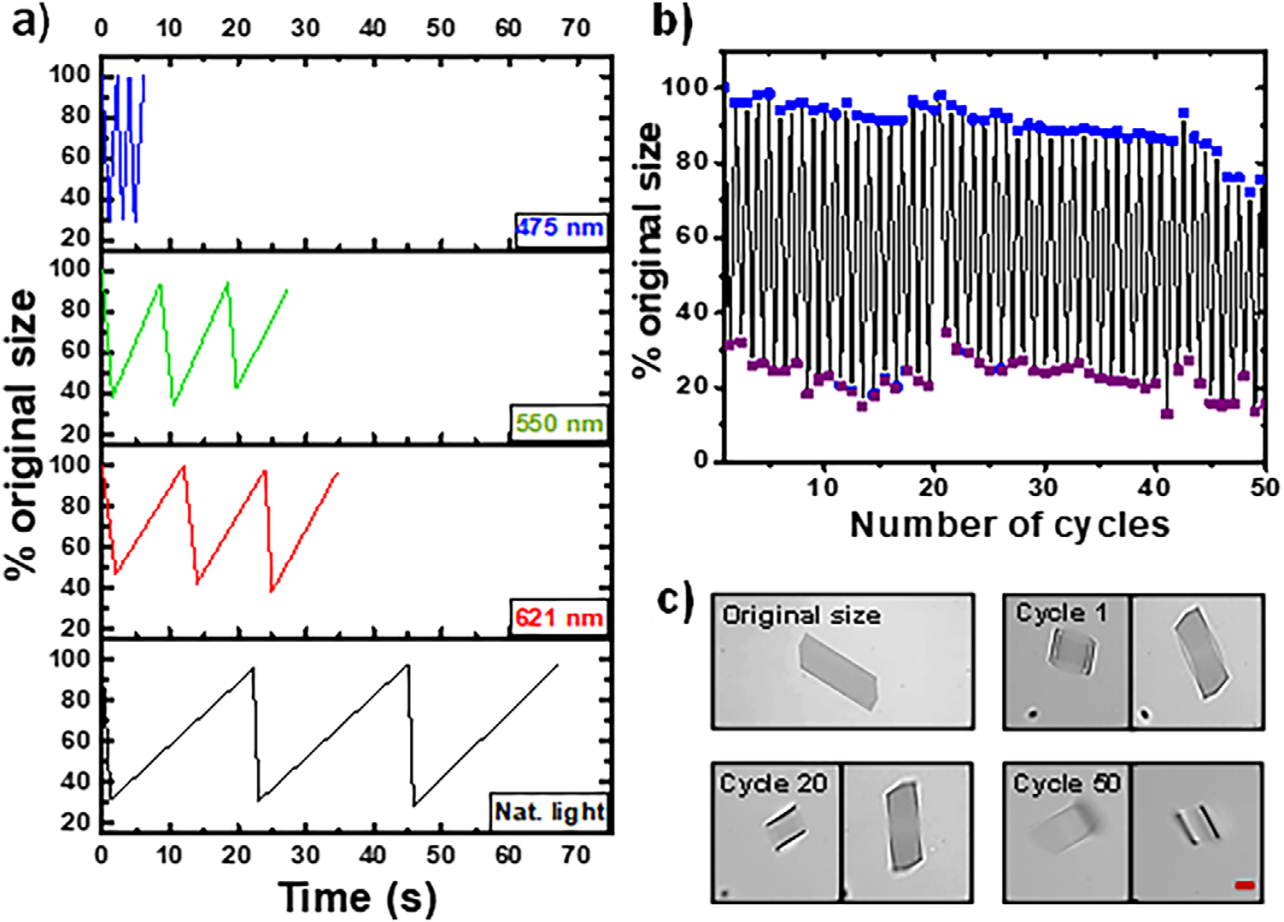
a) Time‐dependence of the relaxation process, with 100% of the power of different lights: 475, 550, 621 nm, and exposed to daylight (denoted as Nat. light). b) Size variations over 50 cycles of excitation (385 nm)‐relaxation (475 nm) expressed as percentages in relation to the original size of the crystals. c) Micrographs taken during different cycles from Video S4 (note the 5‐min video duration corresponding to 50 cycles with 2 s per cycle), during the excitation process (left) and after the relaxation process (right). Scale bar indicates 20 µm.

The bending of α‐*S8* MCs induced by 10% intensity of 385‐nm radiation was subjected to three subsequent excitation–relaxation cycles. However, instead of allowing passive relaxation in the dark, the crystals were exposed to full‐intensity (100%) illumination at three different wavelengths: 475, 550, and 621 nm. As shown in Figure 3a and Video S3, the recovery time exhibits a strong dependence on photon energy, decreasing with shorter wavelengths. Compared to the total of 67 s required for three full cycles under daylight (approximately 22 s per cycle), the same process under 621‐nm light was completed in 35 s (≈12 s per cycle). With 550‐nm illumination, the duration further decreased to 27 s (≈9 s per cycle), and under 475‐nm light, only 6 s were needed for three full cycles (2 s per cycle). These results demonstrate that the recovery of UV‐irradiated bent crystals is significantly accelerated by visible‐light exposure, with shorter wavelengths leading to faster relaxation. This wavelength‐dependent behavior suggests the involvement of a combined photoexcitation–photo‐relaxation mechanism.

Based on these results, 10% of the power of 385 nm (4.7 mW mm^−2^) was selected as the excitation wavelength, while 475 nm, applying 100% of the total power (22.8 mW mm^−2^), was identified as the most effective radiation for relaxation. With these parameters fixed, and to further characterize the behavior of the α‐*S8* MCs cycling repeatedly, one crystal was submitted to several cycles (Figure 3b,c and Video S4); after 50 cycles of excitation‐relaxation, the α‐*S8* MC retained its overall shape with no significant changes, exhibiting only a slight curvature. On average, the bending deformation reached 22 ± 5% of the original size, while the recovery efficiency was measured at 90 ± 6%. After 52 cycles, the α‐*S8* MC remained stuck in the bent form. The same experiment was repeated with 10 α‐*S8* MCs of identical shapes, showing an average bending deformation of 20 ± 10% within 0.7 ± 0.2 s (at 385‐nm) and a recovery efficiency of 89 ± 5% within 1.0 ± 0.4 s (at 475‐nm). The maximum number of cycles varied widely due to several factors: changes in the crystal positions caused by repeated excitation–relaxation movements, which sometimes exposed only an edge and reduced the interaction with radiation; irreversible bending; degradation resulting from increased sensitivity to radiation; or differences in crystal thickness. However, the crystals consistently withstood at least 10 cycles.

**Figure 4:**
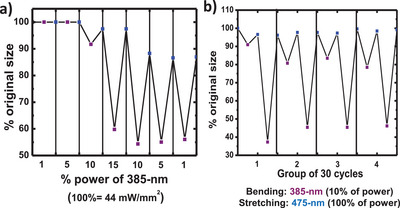
a) Percentage of power (100% of power = 44 mW mm^−2^) required to bend a single microcrystal. Once the effect has been achieved for the first time, a fraction of the initial energy is able to observe the same effect again. b) First and last size variation after 30 cycles applying 15% of 385‐nm radiation for bending and 100% 475‐nm radiation for stretching, four times, with a break of 1 min between one group and another one.

Interestingly, a sensitization effect was observed, wherein the initial bending of α‐*S8* MCs required relatively high‐intensity 385‐nm irradiation, but subsequent bending cycles could be triggered with much lower intensities. To explore this behavior, a single crystal was subjected to repeated excitation–relaxation cycles under varying 385‐nm intensities, while keeping the 475‐nm recovery light at full intensity throughout (Figure 4a). At low 385‐nm intensities (1%–5%), no deformation was observed. Bending initiated at 10%, reducing the crystal length to ∼92%, and became most pronounced at 15% of the power of the radiation, with shrinkage to 60% of the original size. Notably, after reducing the intensity back to 10%, the crystal continued to bend, shrinking further to 55% of its original size, and remained at that length even when the intensity of the light was reduced to 5% and 1%. This suggests that once an energy barrier is surpassed during the initial high‐power excitation, the system enters a sensitized state that need lower power intensity in subsequent cycles. This sensitized state is only partially relaxed by visible‐light irradiation (475 nm), which is sufficient to restore the crystal's shape but not its full molecular order. Consequently, during this metastable period, even minimal 385‐nm excitation is enough to induce bending again.

To confirm this hypothesis, the crystal was subjected four times to 30 excitation–relaxation cycles, with 1‐min 475‐nm exposure intervals between sets. As shown in Figure 4b, each new cycle set began with weaker bending than the end of the previous one, indicating gradual relaxation back to the ground state. This supports the existence of a transient sensitized state and an energy barrier that governs the bending response.

The potential influence of crystal morphology on the bending behavior was examined by subjecting needle‐like α‐*S8* crystals to excitation–relaxation cycling tests. This type of shape of α‐*S8* MCs demonstrated the highest reversibility, enduring up to 200 cycles without any observable change in behavior (see Figure S3 and Video S5; note the 17‐min video duration that covers the 200 cycles). Additionally, the needle‐like α‐*S8* MCs exhibited less bending compared to the previously tested rectangular crystals, with a bending percentage of 48 ± 10%, but showed excellent recovery performance at 93 ± 6%. The excitation and relaxation times were also significantly shorter, averaging 0.5 ± 0.1 and 0.4 ± 0.1 s, respectively. α‐*S8* MCs with other shapes displayed the same behavior as the rectangular one with two truncated opposite squares shown by the initially studied α‐*S8* MCs, as illustrated in Figure S4 and Video S6.

To understand the photo‐responsive properties of α‐*S8* MCs, quantum‐mechanical calculations were carried out. We note that the absorption spectra of α‐*S8* rings in both solution and the solid state are characterized by a strong band at about 300 nm (4.1 eV), followed by a tail extending up to 400 nm (3.1 eV).^[^ ^32^ ^]^ According to time‐dependent density functional theory (TD‐DFT) and highly correlated electronic‐structure methods (such as diagrammatic construction scheme to the second order (ADC(2)) and STEOM‐CCSD), the first singlet excited state (S_1_) of the isolated *S8* molecule is located at about 4.0 eV, a value in good agreement with the value of 3.8 eV estimated from experimental data.^[^ ^32^, ^33^ ^]^ Interestingly, the S_0_→S_1_ transition has a negligible oscillator strength, which explains why *S8* molecules are nonemissive. Also, it must be emphasized that the 385‐nm radiation lies in an energy region below the *S8* singlet states and should therefore be associated with electronic transitions from the ground state to triplet states (S_0_→T_n_). Our earlier electronic‐structure calculations have shown that *S8* rings exhibit significant spin‐orbit coupling, which allows triplet states to become moderately optically active by mixing with singlet states through spin‐orbit interactions.^[^ ^32^ ^]^ According to these calculations, the lowest triplet state is located at about 3.2–3.5 eV.^[^ ^33^, ^34^ ^]^ This rationalizes that the 621‐nm (2.00 eV), 550‐nm (2.25 eV), and 475‐nm (2.61 eV) radiations have no effect when applied to *S8* MCs, as discussed above.

**Figure 5:**
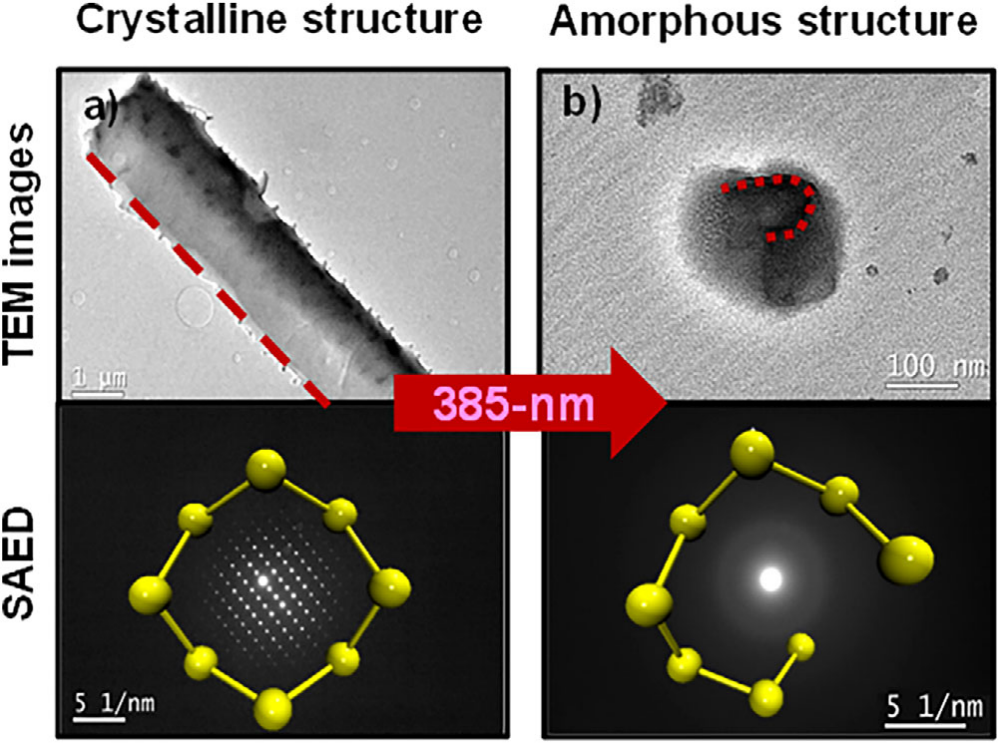
TEM images (top) and electron diffraction pattern (bottom) of α‐*S8* MCs before a) and after b) the exposure to 385‐nm radiation. A model of the sulfur ring is given in yellow (bottom).

Molecular bending in photo‐actuators typically occurs due to photoinduced structural changes, such as *cis*‐*trans* isomerization in azopolymers.^[^ ^35^ ^]^ For *S8* rings, it is well established that laser illumination can induce polymerization below the usual polymerization temperature (∼140 °C) by breaking sulfur–sulfur bonds within the rings.^[^ ^36^, ^37^ ^]^ Indeed, our electronic‐structure calculations based on TD‐DFT underlined that, upon photoexcitation, the *S8* molecules evolve from a ring configuration to an open‐chain configuration that carries a diradical character.^[^ ^34^ ^]^ Moreover, a very recent study based on advanced nonadiabatic dynamics simulations showed that bond breaking within the *S8* rings actually occurs within tens of femtoseconds for both singlet and triplet states.^[^ ^33^ ^]^ Taken together, these results suggest that upon illumination with a 385‐nm radiation, there is a formation of *S8* chains within the MCs, which leads to the emergence of a nonuniform ring‐chain composition. These structural modifications consequently could result in the modification of the mechanical (elastic) properties across the MC films, leading to MCs bending. When the irradiation stops, the *S8* chains are expected to relax back to the ring configurations that are energetically more stable. This is consistent with the experimental data obtained by selected‐area electron diffraction (SAED) performed before (Figure 5a) and after (Figure 5b) irradiation of α‐*S8* MCs with 385‐nm light. Prior to irradiation, the crystals display a well‐defined diffraction pattern characteristic of a highly ordered structure. Following UV exposure, the sharp diffraction spots disappear and are replaced by diffuse halos, indicative of a transition to an amorphous phase.

**Figure 6:**
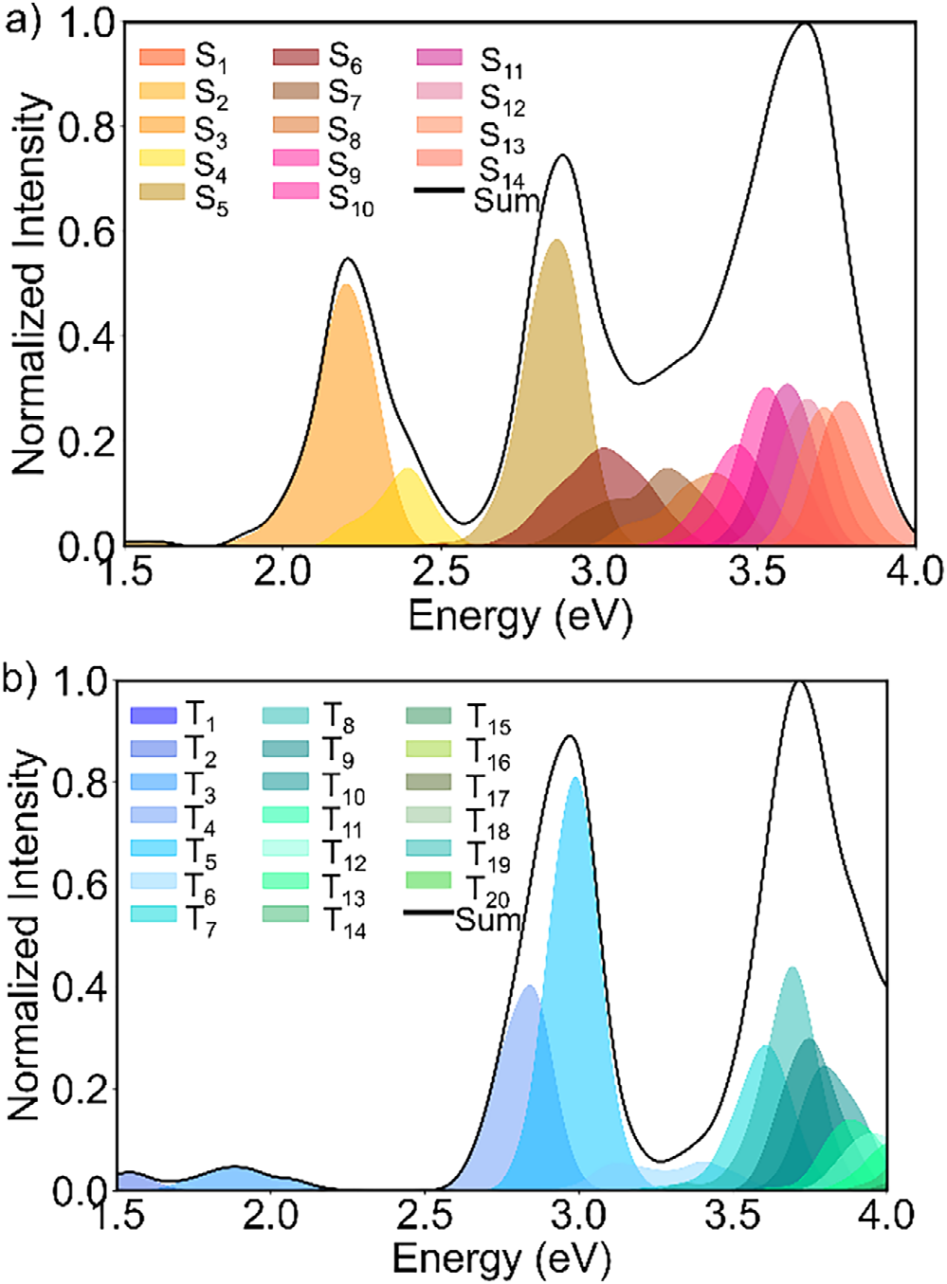
Absorption spectra of *S8* chains for transitions starting from the a) S_0_ and b) T_1_ states.

The remaining question is to rationalize the recovery‐time dependence on the applied light with different wavelengths. To get insight into this issue, we investigated the absorption spectrum of the *S8* chains. Starting from the optimized T_1_ geometry, we performed a Wigner sampling to obtain 500 chain geometries. The absorption spectra from both the S_0_ and T_1_ states of the chains were then calculated with the ADC(2)/def2‐TZVP approach, see Figure 6.

**Figure 7:**
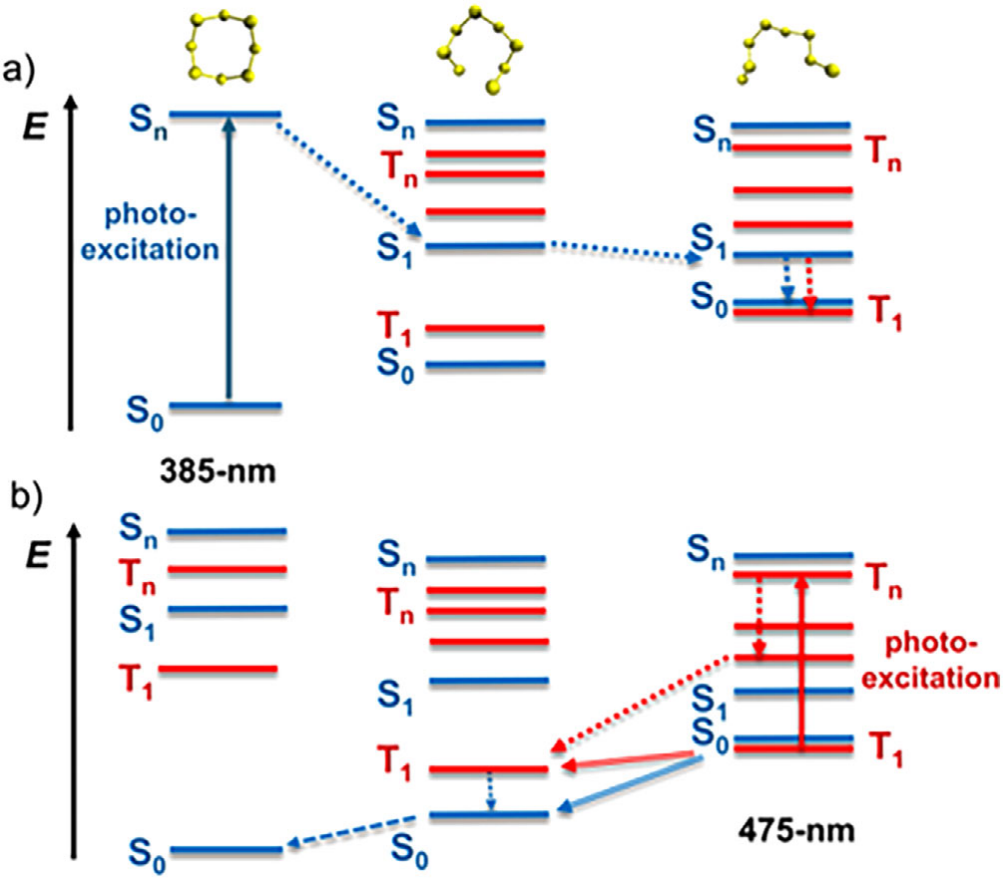
Photo‐excitation pathways for a) ring‐to‐chain and b) chain‐to‐ring transformations.

As seen from Figure 6, the intensity of the electronic transitions increases with energy (i.e., with a decrease in light wavelength). This simply means that photons with higher energy have larger probabilities to promote an *S8* chain to excited states, from which it has a larger probability to relax back to a ring configuration than when starting from the (quasi‐degenerate) S_0_ and T_1_ states of the chain. The reason is that higher‐energy excited *S8* chains can more easily overcome the energy barrier required for the chain‐to‐ring transformation. The related photo‐excitation pathways for ring‐to‐chain and chain‐to‐ring transitions are depicted in Figure 7.

We also evaluated the physicochemical properties and stability of α‐*S8* MCs. The structures were stored in a water solution at pH = 9 (due to the presence of hydrazine) at room temperature and under environmental light; experiments were conducted under these conditions. The α‐*S8* MCs begin to lose integrity and become wrinkled after 2 weeks of storage (Figure S5a). Despite this evolution, their properties remain intact, although a loss of flexibility is observed, requiring increased time and power to achieve a similar extent of bending.

The pH range in which the α‐*S8* MCs can operate was tested from 0 (1 M HCl) to 14 (1 M NaOH), both in aqueous medium. The results show that their properties are maintained across this pH range, with the integrity of the α‐*S8* MCs remaining unaffected up to 30 min under the most extreme conditions (Figure S5b,c). The role of the solvent was also investigated by depositing two drops of α‐*S8* MCs dispersion onto a glass slide. After a few minutes, when most of the α‐*S8* MCs had settled at the bottom of the drop, the solvent was removed using a micropipette and replaced with DMF and DMSO, respectively. Upon irradiation, the α‐*S8* MCs exhibited the same behavior: bending of the crystal under 385‐nm radiation and rapid recovery of the original shape with 475‐nm light, as shown in Figure S5d. Notably, gradual dissolution of the α‐*S8* MCs in both DMF and DMSO was observed over time.

The bending effect was also tested in a dry environment. A water dispersion of α‐*S8* MCs was deposited on a glass slide, and, after water evaporation, the crystals were exposed to 385‐nm radiation. Here, the previously observed effect did not occur at any intensity of the 385‐nm LED. Instead, after 5–10 s at maximum power, the α‐*S8* MCs began to melt irreversibly. This outcome is attributed to two factors: the inability to dissipate heat due to the absence of water, leading to melting, and the high surface‐to‐thickness ratio of the crystals. Once the water evaporates, the α‐*S8* MCs start strongly adhering to the glass surface, preventing them from bending. However, after rehydration, the effect is fully restored.

The last tests focused on the evaluation of the role of temperature in the irradiation process. On the one hand, the evolution of the temperature was measured in a drop of water with α‐*S8* MCs during the irradiation with 385‐nm light for 10 min with 100% of power (44 mW mm^−2^), in comparison to a drop of just distilled water under the same conditions. The results show that the increase in temperature at that time is totally comparable in both cases, around 5 °C. The highest temperature was 32 °C (Figure S6a), which is not high enough to provoke any melting process in the α‐*S8* MCs. Thus, the irradiation was not causing any significant increase in the temperature of the medium. On the other hand, a drop of water dispersion of α‐*S8* MCs was placed on a glass slide without any kind of irradiation. When almost all the water was evaporated but with some remaining water to prevent the α‐*S8* MCs from sticking on the glass surface, some drops of boiling water were added where the α‐*S8* MCs were located. In this case, no bending effect was observed (Video S7); the only notable phenomenon was the partial melt of some α‐*S8* MCs (Figure S6b) because of the high water temperature. These two tests demonstrate that the temperature in the irradiation process and even the partial melting of the crystals by thermal effect do not have a relevant role in the bending effect of the α‐*S8* MCs.

## Conclusion

We have unveiled the photo‐responsive properties of α‐*S8* MCs, showcasing their remarkable ability to bend upon 385‐nm light irradiation; the shape recovery is triggered by sequential irradiation with 475‐nm light. These fascinating muscle‐like properties are totally independent of the shape and size. The underlying mechanism involves a combination of ring‐to‐chain and chain‐to‐ring transformations: the breaking of *S8* rings induces bending in the α‐*S8* MCs, while the excitation of the chains facilitates an accelerated relaxation process, allowing the material to swiftly regain its ring shape. This interplay of processes reveals a remarkable property of inorganic materials, establishing these α‐*S8* MCs as the simplest known actuators of their kind to date. Given their capabilities, α‐*S8* MCs hold significant potential for applications in micro‐ and nanomanipulation, sensor‐actuators, as well as in the development of intelligent gating systems.

## Conflict of Interests

The authors declare no conflict of interest.

## Supporting information



Supporting Information

Supporting Information

Supporting Information

Supporting Information

Supporting Information

Supporting Information

Supporting Information

Supporting Information

## Data Availability

The data that support the findings of this study are available on request from the corresponding author. The data are not publicly available due to privacy or ethical restrictions.
