# Supplementary material for: Cyclo‐Octasulfur Crystals as Light‐Controlled Molecular Muscles
Source: Angew Chem Int Ed Engl. 2025 Jul 1;64(34):e202506269. doi: 10.1002/anie.202506269 (PMC12363614; doi:10.1002/anie.202506269)
Supplement: Supplementary file 1 — Supporting Information [file ANIE-64-e202506269-s007.docx]

Supporting information

**Cyclo-octasulfur Crystals as Light-controlled Molecular Muscles**

Enrique Solano-Rodríguez, Qi Sun, Jean-Luc Brédas, Veaceslav Coropceanu*, Beatriz Jurado-Sánchez*, and Alberto Escarpa*

**Experimental procedure**

**MATERIALS AND METHODS**

**Reagents and materials.**

Ammonium tetrathiomolybdate, (NH_4_)_2_MoS_4_ (cat. 323446), and hydrazine solution (35 wt. % in water, cat. 309400) were supplied by Sigma-Aldrich (Spain). The stainless-Teflon reactor was made by the Mechanical Workshop of the University Complutense of Madrid (UCM).

**Parameters and reproducibility of the synthesis:**

The reported synthesis is totally reproducible, growing α-*S8* MCs in all the several attempts. It was evaluated the role of different factors, such as pH, light or darkness, and temperature during synthesis.

Evaluation of the role of the pH: One synthesis was subdivided into different vials, changing the pH of the medium from 1 to 14. In the acid mediums a quick precipitation of the MoS_2_ was observed, giving consequently a totally colorless and clear supernatant. In those cases, no α-*S8* MCs were found. In the basic medium, on the other hand, it was detected α-*S8* MCs in all cases, giving more product when the medium was more basic, until the stabilization in the pH = 11.

Evaluation of the role of the light: The previous experiments were carried out in darkness, but we obtained the same results when exposed to light. It can be said light has not a significant role in the growth of α-*S8* MCs.

Evaluation of the role of the temperature: We investigated two temperature-related aspects: the temperature during synthesis and the temperature during crystallization.

On the one hand, different synthesis temperatures were tested by preparing batches from room temperature up to 200 °C. Between 100 °C and 200 °C, no significant differences were observed: in all cases, a black liquid was obtained, from which small α-*S8* MCs became visible after two days. However, when the synthesis temperature was lower than 100 °C, the resulting solution remained orange, with no observable precipitation or crystallization.

On the other hand, we examined the effect of crystallization temperature. A sample synthesized using a reported method (10 h at 110 °C) was divided into three portions and stored under different conditions: in a refrigerator, at room temperature, and in an incubator at 37 °C. In all cases, α-*S8* MCs formed without significant variation in crystallization time, indicating that the crystallization temperature is not a critical factor within this range.

The obtained products have the same shape and size in all cases. The major part (~95%) of the α-*S8* MCs presented rectangle with two truncated opposite squares. Only a small fraction of the crystals presented different shapes like needle or hexagons.

**Equipment.**

To record the videos and to excite the micro-crystals, a Nikon Eclipse Ti-2 inverted optical microscope was used, coupled to a PCO. A panda camera and a multi-LED light illumination source, model Nikon D-LEDI-C, were used to excite and de-excite the MCs. TEM images, EDX elemental analysis, and electron diffraction were carried out with a JEOL-JEM 2100 model at the Spanish National Electronic Microscopy Centre. XRD diffraction patterns were obtained with a Bruker D8 Advance A25 diffractometer from the X-ray unit at the University Complutense of Madrid. Raman spectra were collected in the “Spectroscopy and co-relation unit” from the University Complutense of Madrid.

**Computational methodology.**

The simulation of the absorption spectra of *S8* chains in the singlet and triplet states is conducted at ADC(2)/def2-TZVP level of theory with the TURBOMOLE ^[1]^ ^[2]^ code in conjunction with the SHARC software^[3]^ ^[4]^

**Supplementary scheme and figures**

**Scheme S1**: Synthetic route of α-*S8* MCs

**Figure S1**: (a) Raman spectra of sulfur nanoparticles (red) and bibliographical α-*S8* sulfur (black) ^[5]^. (b) Optical image of sulfur nanoparticles.

**Figure S2**: Explanation of size variation measurement: The percentage of bending is calculated as the ratio of the bent length to the initial length. Similarly, the percentage of recovery is determined by the ratio of the recovered length to the initial length.


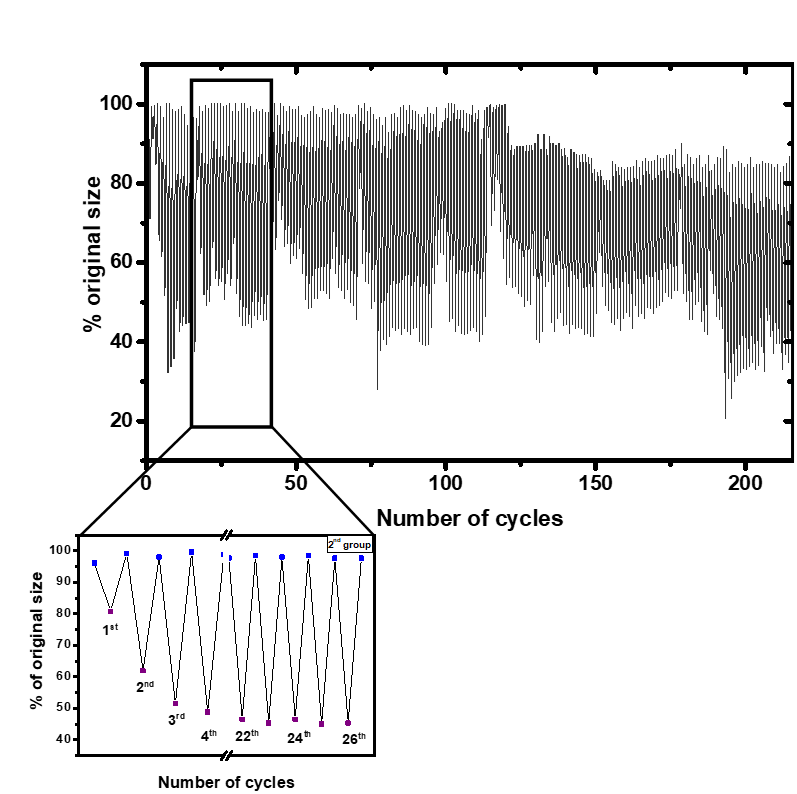


**Figure S3.** Size variations over two hundred cycles of excitation-relaxation cycles in groups of approximately thirty cycles, expressed as a percentage relative to the original size of a needle-like crystal, taken from **video S5**, and the enlargement of the second group, where it can be seen how the size variation is maintained from the 4^th^ cycle to the 30^th^ cycle.

­­**Figure S4**. Different morphologies reacting under 385 nm radiation.

**Figure S5.** Aspect of α-*S8* MCs after two weeks of storage at room temperature and pH=9 under natural light (a). Behaviour of MCs under pH=0 (b) and pH=14 (c) from 0 minutes (top) to 30 minutes (bottom). (d) Behaviour of MCs before (top) and after (bottom) irradiation with 385 nm light using DMF as solvent. Scale bar: 20 µm.

**Figure S6**. (a) Temperature during the irradiation with 385-nm, with 100% of power (44 mW·mm^2^), of α-*S8* MCs in water (yellow) and distillated water for 10 minutes. It can be seen how the increase of temperature is totally comparable in both cases, ruling out the photothermal effect. (b) Effect of boiling water in the α-*S8* MCs, provoking a partial melting but not a bending effect.

**Supplementary videos**

**Video S1:** *Set up excitation α-S8 MCs with the microscope.* This video demonstrates how the α-*S8* MCs sample is positioned under the microscope and sequentially irradiated, first with 385-nm light, followed by 475-nm light.

**Video S2:** *Behavior of α-S8 MCs irradiated at different wavelengths during one minute:* In this video, it shows the response of α-*S8* MCs under different wavelengths: 385, 475, 550, and 621 nm. A noticeable reaction occurs only under 385 nm irradiation, where the MCs begin to bend immediately within 2 seconds, even at just 10% of the LED's power. As irradiation continues, the bending progresses at a relatively slow rate. After 30 seconds, when the LED power increases to 100%, the MCs exhibit a dramatic bending response, suggesting a significant change in their physical properties. In contrast, exposure to the other wavelengths does not induce any noticeable response, even at full power and after 60 seconds of irradiation.

**Video S3:** *Recovery time-dependence at different wavelengths*. This video shows the different times that take the recovery after the bending provoked by 385 nm radiation, under three different wavelengths: 475, 550, and 621 nm. It can be noticed that the recovery rate is directly related to the energy of the light, the lower is the wavelength, the faster the recovery to the original shape.

**Video S4:** *Cycling α-S8 MC 50 times*. This video presents a rectangle with two truncated opposite squares α-*S8* MCs cycling 50 times, under 385 nm radiation for the bending, and under 475 nm radiation for the recovery.

**Video S5:** *Needle-like α-S8 MCs cycling 200 times*. This video presents a needle-like α-*S8* MCs cycling 200 times, under 385 nm radiation for the bending, and under 475 nm radiation for the recovery.

**Video S6*:*** *Different α-S8 MC´s shapes cycling*. This video demonstrates that the observed effect is independent of the morphology of the α-*S8* MCs, as the same phenomenon occurs in four different crystal shapes.

**Video S7:** *Temperature control: effect of high temperature on α-S8 MCs.* In this video, no bending effect is observed in the α-*S8* MCs upon exposure to boiling water. This indicates that the bending is not thermally induced but rather arises from a photoelectronic mechanism.

**Supporting References**

[1] Y. J. Franzke, C. Holzer, J. H. Andersen, T. Begušić, F. Bruder, S. Coriani, F. Della Sala, E. Fabiano, D. A. Fedotov, S. Fürst, S. Gillhuber, R. Grotjahn, M. Kaupp, M. Kehry, M. Krstić, F. Mack, S. Majumdar, B. D. Nguyen, S. M. Parker, F. Pauly, A. Pausch, E. Perlt, G. S. Phun, A. Rajabi, D. Rappoport, B. Samal, T. Schrader, M. Sharma, E. Tapavicza, R. S. Treß, V. Voora, A. Wodyński, J. M. Yu, B. Zerulla, F. Furche, C. Hättig, M. Sierka, D. P. Tew, F. Weigend, *Journal of Chemical Theory and Computation* **2023**, *19*, 6859-6890.

[2] F. Furche, R. Ahlrichs, C. Hättig, W. Klopper, M. Sierka, F. Weigend, *WIREs Computational Molecular Science* **2014**, *4*, 91-100.

[3] S. Mai, P. Marquetand, L. González, *WIREs Computational Molecular Science* **2018**, *8*, e1370.

[4] M. Richter, P. Marquetand, J. González-Vázquez, I. Sola, L. González, *Journal of Chemical Theory and Computation* **2011**, *7*, 1253-1258.

[5] C. Nims, B. Cron, M. Wetherington, J. Macalady, J. Cosmidis, *Scientific Reports* **2019**, *9*, 7971.
